# Impact of audible pops associated with spinal manipulation on perceived pain: a systematic review

**DOI:** 10.1186/s12998-022-00454-0

**Published:** 2022-10-04

**Authors:** Annelieke Cesanne Moorman, David Newell

**Affiliations:** 1 AECC University College, Bournemouth, UK; 2 Private Practice, Maastricht, The Netherlands

**Keywords:** Spinal manipulation, Audible pop, Pain, Systematic review, Chiropractic

## Abstract

**Objectives:**

An audible pop is the sound that can derive from an adjustment in spinal manipulative therapy and is often seen as an indicator of a successful treatment. A review conducted in 1998 concluded that there was little scientific evidence to support any therapeutic benefit derived from the audible pop. Since then, research methods have evolved considerably creating opportunities for new evidence to emerge. It was therefore timely to review the evidence.

**Methods:**

The following electronic databases were searched for relevant studies pertaining to the impact of audible pops in spinal manipulative therapy: PubMed, Index to Chiropractic Literature (ICL), Cumulative Index to Nursing & Allied Health Literature (CINAHL) and Web-of-Science. The main outcome was pain. Two reviewers independently selected studies, extracted data, and assessed risk of bias and quality of the evidence using the Downs and Black checklist. Results of the included literature were synthesized into a systematic review.

**Results:**

Five original research articles were included in the review, of which four were prospective cohort studies and one a randomized controlled trial. All studies reported similar results: regardless of the area of the spine manipulated or follow-up time, there was no evidence of improved pain outcomes associated with an audible pop. One study even reported a hypoalgesic effect to external pain stimuli after spinal manipulation, regardless of an audible pop.

**Conclusions:**

Whilst there is still no consensus among chiropractors on the association of an audible pop and pain outcomes in spinal manipulative therapy, knowledge about the audible pop has advanced. This review suggests that the presence or absence of an audible pop may not be important regarding pain outcomes with spinal manipulation.

**Supplementary Information:**

The online version contains supplementary material available at 10.1186/s12998-022-00454-0.

## Background

Spinal pain, particularly in the lumbar and cervical region, is an extremely common health problem worldwide. In addition to being of considerable impact on an individual’s life it brings a considerable societal cost often resulting in job-related disability as a leading contributor to missed workdays [1]. Manual therapeutic approaches are widely used by patients with spinal pain, with about 75% of patients consulting either chiropractic care, physical therapy or osteopathy [2]. Such clinicians commonly offer a package of care, which can include multiple modalities with the common addition of spinal manipulative therapy (SMT) and/or spinal mobilization as treatment options. These packages of care are presently recommended as management options in practice guidelines for the management of both low back pain (LBP) and neck pain [3–5].

Many chiropractic patients are familiar with hearing a popping or cracking sound when receiving SMT and this is often seen as a factor that differentiates mobilization and manipulation [6]. To the clinician delivering SMT, this sound is frequently associated with the perception of a successful intervention [6] and when it does not occur, some clinicians may apply another treatment thrust [7].

Typically, with high-velocity low-amplitude (HVLA) SMT the target joint is brought to its end range of motion (ROM) as the practitioner applies a directed preload force followed by a thrust. The force delivered takes the joint beyond its regular end range of motion, allowing the joint to move into the para-physiological movement zone [8]. Current evidence by Kawchuck et al. [9] supports ‘tribonucleation’ as the process inducing the joint to generate a cracking sound. When sufficient distraction force overcomes the viscous attraction or adhesive forces between opposing joint surfaces, rapid separation of the articulation occurs with the resulting drop in synovial pressure allowing dissolved gas to come out of solution to form a cavity within the joint. This cavity persists after the popping sound is produced and therefore proposes that joint cracking is associated with cavity formation within the synovial fluid, rather than cavity collapse as has been the viewpoint for many years [10]. More recently, Suja and Barakat [11] also noted that tribonucleation is the most likely triggering mechanism for cavitation in the synovial fluid, supporting Kawchuck et al. [9].

Whilst historical theories see SMT as primarily aiming to restore joint function and mobility, the exact mechanisms by which it achieves this, or whether such interventions are responsible for the documented decreases in pain and improvements in function [12], remain disputed or unknown. Other mechanisms invoking psychological reassurance from personal interaction and therapeutic touch by the clinician associated with inhibition of ascending and/or descending sensory neural pathways or reflex changes have been proposed [13, 14]. Indeed, Herzog [15] suggests that SMT produces reflex responses in muscle tone, with effects reaching locations that are distant to the treatment site.

One of the effects of an audible pop (AP) is believed to be its contribution to these muscle reflex responses [10]. However, such a mechanism was countered by Herzog [15], who showed that an AP can be elicited with a slow force application and is not associated with a corresponding electromyography response. This suggests that the AP may not be responsible for the reflex responses observed during SMT [16]. Whilst the AP is inextricably associated with SMT, there is currently no consensus on its clinical relevance.

This review was conducted to assess and update the evidence pertaining to the potential role of the AP in obtaining therapeutic benefits associated with SMT, specifically if the AP plays a role in decreasing pain perception. This is key to understand the mechanisms behind SMT associated clinical benefits and may help to inform strategies to improve the effectiveness of the treatment.

## Method

This review follows the guidelines for Preferred Reporting Items for Systematic reviews and Meta-analyses (PRISMA) [17] as shown in Additional file 1: Table S1. Its protocol is registered with the International Prospective Register of Systematic Reviews (PROSPERO) (reference CRD42021259716).

### Search strategy

The following electronic databases were searched: PubMed, Index to Chiropractic Literature (ICL), Cumulative Index to Nursing & Allied Health Literature (CINAHL) and Web-of-Science. The search was first conducted in March 2020 and an updated search was conducted in June 2022. The search terms “audible release/pop”, “joint cavitation/cracking”, “spinal manipulative therapy”, “chiropractic adjustment/manipulation”, “high velocity low amplitude adjustment”, “spinal manipulation” and “pain” were used individually and jointly. MeSH terms were applied, where available, and reference lists of the included studies were screened allowing all relevant papers to be sourced that included alternative terms. The detailed search strategy for CINAHL is shown in Additional file 2: Table S2.

### Eligibility criteria for study inclusion and exclusion

*Inclusion criteria were as follows*: Empirical and mixed-method studies, studies published in the English language, human participants, adults (over 18 years old), studies aiming to assess pain outcomes following the occurrence of an AP associated with SMT, exposure consisting of spinal (cervical, thoracic, lumbar) or lumbo-pelvic adjustment(s). Both symptomatic and asymptomatic participants were included. Only peer-reviewed studies were included in order to assure exclusion of low-quality studies.

*Exclusion criteria were as follows*: Letters, dissertations, commentaries, editorials, conference abstracts, studies published in language other than English, animal participants, children (under 18 years old), studies not aiming to assess pain outcomes, exposure consisting of extremity adjustment(s).

### Screening and selection

Systematic screening was performed following the steps of the PRISMA [17] protocol. Duplicates were removed electronically by uploading the titles into Rayyan software [18]. Consensus was reached during meetings of both authors. The remaining titles and abstracts were independently screened by both authors. If determination could not be made based on the abstract, the full-text was reviewed for inclusion and exclusion criteria.

### Quality assessment

Each included study was assessed using the Downs and Black [19] checklist. This checklist includes 27 criteria, widely covering areas reporting quality, external and internal validity, and power. The quality of each study was independently assessed by both authors, with discrepancies resolved through meetings.

## Results

**Fig. 1:**
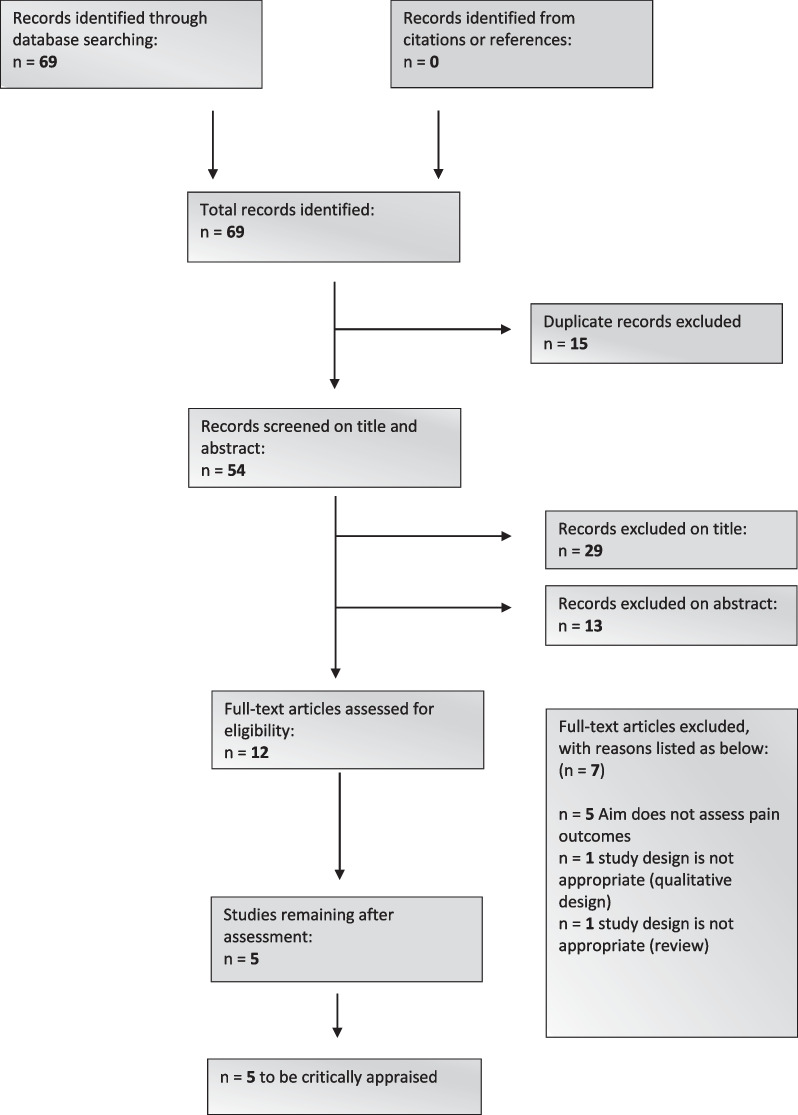
PRISMA flow diagram

Of the initial 69 studies obtained, five were included in this review as illustrated in Fig. 1.

### General study characteristics

The study characteristics of the five included studies are summarized in Table 1. All studies aimed to investigate the effect of an AP during SMT on pain outcomes. Most assessed the influence of the intervention on existing musculoskeletal pain. Bialosky et al. [20] took a different approach by applying the intervention on healthy subjects followed by an external thermal pain stimulus to assess outcomes. One included study [21] was an RCT, whereas the other studies were prospective cohorts [20, 22–24]. Four out of the five studies [20–23] are secondary analyses of which the protocol and primary results are provided in detail elsewhere. All studies were carried out in the U.S.A.

**Table 1:** Study characteristics

Study	Aim	Participants	Intervention	Presence of AP’S	Outcome measure	Conclusion
Bialosky et al. [20] U.S.A Prospective Cohort Secondary analysis	Assess the role of the AP in HVLA manipulation associated thermal pain sensitivity to both A-delta fiber mediated pain perception and temporal summation	n = 40 Males and females, pain free, aged 18–60	HVLA thrust technique on the sacroiliac joint two times on each side, regardless of whether an AP was perceived. The examiner noted if AP was perceived	AP present: n = 18 AP absent: n = 22	NPRS as a measure of expected and perceived pain and psychological questionnaires (PCS, FPQ-III and anxiety VAS). Immediate follow-up directly after intervention and after 4 min	Hypoalgesia is associated with HVLA manipulation and occurs independently of a perceived AP. Inhibition of lower extremity temporal summation may be larger when AP is perceived
Sillevis and Cleland [21] U.S.A RCT secondary analysis	1. Determine if audible sounds during a thrust manipulation have an immediate effect on pain perception within a group of chronic neck pain patients. 2. Determine if there is a positive correlation between the presence of audible sounds and a change in autonomic function	n = 100 Males and females with chronic (present for at least 3 months) cervical pain aged 18–65	High-velocity anterior-to-posterior force to the upper thoracic spine targeting T3-T4 segment in supine position. Presence of audible sounds recorded by the researcher. Control: mobilization directed at T3-T4	n = 50 mobilization n = 50 manipulation of which One pop: n = 14 Multiple pops: n = 18 No pop: n = 18	VAS used to assess pain and automated pupillometry used to capture pupil responsiveness measured in the form of pupil diameter. Follow-up directly after intervention and after 4 min	The presence of joint sounds does not influence the overall activity of the autonomic nervous system (P = .31, .44, .47, respectively) following a thrust manipulation or contribute to the reduction of pain (P > 0.05) in patients with chronic neck pain
Cleland et al. [22] U.S.A Prospective Cohort secondary analysis	Examine the relationship between the audible pop and patient-centered outcomes in a cohort of patients with neck pain treated with thoracic spine thrust manipulation	n = 72 Males and females with neck pain with or without unilateral upper extremity symptoms, and baseline NDI > 10% aged 18–60	3 different thrust manipulation techniques to the thoracic spine: distraction manipulation, upper and middle thoracic spine manipulation. The presence or absence of an AP was noted by the treating therapist. 3 thrusts were repeated if no AP was noted	≤ 3 pops: n = 21 > 3 pops: n = 51	Change scores for pain and disability (NDI and NPRS) and cervical ROM. Additionally, GROC was completed. Follow-up 2–4 days after first session	No relationship between the number of audible pops and clinically meaningful improvements in pain (P = 0.41), disability (P = 0.66) or cervical ROM (P > 0.05) in patients with neck pain
Flynn et al. [23] U.S.A Prospective Cohort secondary analysis	Investigate whether the occurrence of a manipulative pop during sacroiliac region manipulation is related to the outcome of the intervention over a 4-week period	n = 70 Males and females with a primary complaint of low back pain with or without referral into the lower extremity and an ODQ score of > 30% aged 18–60	Total of 5 physical therapy sessions over 4 weeks. In the first two sessions pts received HVLA thrust manipulation on the sacroiliac region on the more symptomatic side, and ROM exercise. If cavitation heard onto ROM exercise. If no cavitation heard after additional attempt on opposite side with a maximum of two attempts per side, onto ROM exercise. Cavitation was noted by the patient or therapist	AP present: n = 59 AP absent: n = 11	NPRS to rate pain intensity, ODQ and measurement of lumbopelvic flexion ROM changes. Follow-up at 1 week, and 4 weeks after intervention	AP may not relate to improved outcomes from HVLA thrust manipulation for patients with non-radicular low back pain at either an immediate odds ratio: 1.1 (95% CI, 0.29–3.86) or longer-term odds ratio: 1.7 (95% CI, 0.41–7.1) follow-up
Flynn et al. [24] U.S.A Prospective Cohort	Investigate whether the occurrence of a manipulative pop during sacroiliac region manipulation is related to the outcome of the intervention	n = 71 Males and females with non-radicular low back pain aged 18–60	HVLA thrust technique on the sacroiliac joint, side determined by an algorithm. The therapist noted whether the therapist or patient heard an AP. If no AP, the manipulation was repeated. If no AP, the therapist manipulated the opposite site with a maximum of 2 attempts per side	AP present: n = 50 AP absent: n = 21	Changes in ROM, NPRS scores and modified ODQ scores. Reassessment 48 h after the manipulation	No relationship between AP during sacroiliac region manipulation and improvement in ROM (P = 0.74), pain (P = 0.23) or disability (0.49). Occurrence of AP did not improve odds of dramatic improvement

*AP* Audible Pop,* HVLA* High-Velocity Low-Amplitude,* NPRS* Numeric Pain Rating Scale,* PCS* Pain Catastrophizing Scale,* FPQ-III* Fear of Pain Questionnaire III,* VAS* Visual Analog Scale,* RCT* Randomized Controlled Trial,* NDI* Neck Disability Index, *ROM* Range of Motion,* GROC* Global Rating of Change,* ODQ* Oswestry Disability Questionnaire.

### Participants

The included studies resulted in a total of 303 participants. All participants were between the ages of 18 and 65, of which 38.7% male and 61.3% female respectively. Four studies included participants with pain complaints [21–24], one study included healthy and pain free participants [20]. Of the participants with pain complaints, two studies included participants with cervical pain [21, 22]. One of these solely described neck pain [22], whilst the other also included the upper thoracic region until the level of T4 [21]. The remaining two studies included LBP, one with radiation into the lower extremity [23] and one without radiation [24]. Substantive heterogeneity, as judged qualitatively, existed between the five studies as participants were with or without pain, had pain at differing sites and presented with differing durations of pain; however, there were no other significant differences in baseline characteristics of participants in each individual study.

### Interventions

All interventions consisted of SMT. Most were performed in a single session, with the exception of one study [23] that performed five sessions. All SMT interventions documented a high velocity thrust by a skilled practitioner with the site of the adjustment varying dependent on participant pain location. Two studies with LBP participants [23, 25] and one with pain-free participants [20] chose to use sacroiliac region manipulation, while the two studies with neck pain participants [21, 22] used thoracic spine manipulation techniques. If no AP was recorded on the first attempt most studies repeated the SMT with a maximum of four thrusts in total [22–24]. One study [20] applied four thrusts regardless of whether an AP was perceived and only Sillevis and Cleland [21] did not repeat the thrust if an AP was not perceived on the first try.

In the only RCT included [21] all participants were randomly assigned into either the intervention (manipulation of T3-T4) or the control group which consisted of a mobilization technique where the participant was placed in a position similar to the manipulation technique, but with an open-hand placement at T3-T4 level.

### Presence of APs

The determination of an AP occurring was based on the practitioner’s perception of hearing an AP during the application of the technique, which has been reported to be valid [25]. Three articles [22–24] also included the participant’s perception of hearing an AP, and Bialosky et al. [20] included the practitioner’s perception of feeling an AP.

When an AP occurred, this was recorded immediately by the practitioner. It was noted as either ‘pop’ or ‘no-pop’, except for Sillevis and Cleland [21] who added ‘multiple pops’ as a possibility and Cleland et al. [22] who categorized as over or under three pops. Overall, an AP was perceived on 210 participants and an AP was absent on 72 participants. The studies did not provide information on the total of SMT thrusts that were applied. Because Cleland et al. [22] noted over or under three pops, the number categorized as “under three” can mean either no pop or one or two pops. Thus, on 21 participants it is unclear if an AP did or did not occur.

### Outcome measures

All studies used a form of either the Numeric Pain Rating Scale (NPRS) or Visual Analog Scale (VAS) to measure pain and/or anxiety intensity, which were self-completed by the participants. The pain and/or anxiety VAS is a unidimensional measure of pain intensity which is described as the most sensitive single-item measure for clinical pain research [26], the NPRS is a segmented numeric version of the VAS. Additionally, all five studies used varying questionnaires to assess pain beliefs (Pain Catastrophizing Scale, Fear of Pain Questionnaire III) or disability (Neck Disability Index, Oswestry Disability Questionnaire). Three out of the five studies [22–24] used degrees of ROM in addition to the questionnaires. Sillevis and Cleland [21] uniquely used automated pupillometry to capture pupil responsiveness.

The studies differed in their timing of follow-up. Some used immediate follow-up [20, 21], others used a follow-up after two to four days [22, 24]. Flynn et al. [23] had the longest follow-up at one- and four-weeks post treatment.

### Main effects

Regarding any potential relationship between perceived pain and the presence of an AP during SMT all studies concluded that there was no statistically significant association.

No significant difference in follow-up scores existed between groups with AP and without AP as seen in Table 1. Bialosky et al. [20] observed a trend of moderate magnitude that suggested greater hypoalgesia to temporal summation (second pain) in the lower extremity when an AP was received, although this was not significant. Interestingly, Cleland et al. [22] reported a potential inverse relationship finding that patients who experienced three or less APs were 1.3 times more likely to experience a successful outcome than the group that experienced over three APs.

### Evaluation of bias

The results of the risk of bias assessment are reported in Table 2, establishing a judgement on the studies’ results. Using the Downs and Black Checklist [19], the included studies scored between 18 and 22 points for quality, out of a possible 28 points. Following corresponding quality levels as previously reported [27], the included studies were of fair (15–19 points) to good (20–25 points) quality. All studies clearly described the intervention. All samples of participants were assembled at a common point in the course of the participants complaints and internal comparison groups were used. Patient follow-up was sufficiently long for each individual aim of the studies. When follow-up was incomplete, reasons of dropout were stated and did not rate over 20% of the original sample size. None of the studies reported attempts of blinding, though it should be noted that this is not feasible to apply to these study methods. For all studies, this could have given rise to expectation bias by the participant or to assessor bias. All studies used subjective outcome measures to measure pain and/or anxiety. Therefore, outcome measures are reported as high risk of bias. However, there currently exists no validated tool to measure an individual’s pain objectively, thus it is not fair to consider this a quality issue for these studies. Three studies calculated odd’s ratio [22–24] but adequate adjusting for confounding variables was not reported or was unclear.

**Table 2:** Evaluation of Bias

		Bialosky (2010)	Cleland (2007)	Flynn (2003)	Flynn (2006)	Sillevis (2011)
*Reporting*						
Q1	Hypothesis/aim/objective clearly described	1	1	1	1	1
Q2	Main outcomes in Introduction or Methods	1	1	1	1	1
Q3	Patient characheristics clearly described	1	1	1	1	1
Q4	Interventions of interest clearly described	1	1	1	1	1
Q5	Principal confounders clearly described	2	1	1	1	1
Q6	Main findings clearly described	1	1	1	1	1
Q7	Estimates of random variability provided for main outcomes	1	1	1	1	1
Q8	All adverse events of interventon reported	0	0	0	0	0
Q9	Characteristics of patients lost to follow-up described	0	1	1	1	1
Q10	Probability values reported for main outcomes	1	1	1	1	1
*External vailidity*						
Q11	Subjects asked to participate were representive of source population	1	1	1	1	1
Q12	Subjects prepared to participate were representive of source population	1	1	1	1	1
Q13	Location and delivery of study treatment was representative of source population	UTD	UTD	UTD	UTD	UTD
*Internal validity*						
Q14	Study participants blinded to treatment	0	0	0	0	0
Q15	Blinded outcome assessment	0	0	0	0	0
Q16	Any data dredging clearly described	1	1	1	1	1
Q17	Analyses adjust for differing lenths of follow-up	1	1	1	1	1
Q18	Appropriate statistical tests performed	1	1	1	1	1
Q19	Compliance with interventions was reliable	1	1	1	1	1
Q20	Outcome measures were reliable and valid	1	1	1	1	1
Q21	All participants recruited from the same source population	1	1	1	1	1
Q22	All participants recruited over the same time period	1	1	1	1	1
Q23	Participants randomized to treatment(s)	0	0	0	0	1
Q24	Allocation of treatment concealed from investigators and participants	0	0	0	0	1
Q25	Adequate adjustment for confounding	UTD	UTD	UTD	UTD	UTD
Q26	Losses to follow-up taken into account	1	UTD	UTD	UTD	1
*Power*						
Q27	Sufficient power to detect treatment effect at significance level of 0.05	UTD	UTD	UTD	UTD	1
Total		20	18	18	18	22

*UTD* Unable to determine

## Discussion

### Main findings

The main findings of this systematic review suggest moderate evidence that APs generated during the application of SMT are not likely to possess independent therapeutic benefit in their impact on pain outcomes. To the authors knowledge, this review is only the second to have evaluated the potential association of APs during SMT in relation to any therapeutic benefit, the first now being over twenty years old [28]. However, this early review located only two studies of which one was a pilot study [29] associated with a full study also reported in the same review [30]. The purpose of the studies included in this early review was to investigate electromyographic (EMG) reflex responses in SMT with and without the AP and did not investigate pain outcomes, hence these studies are excluded from this present review. Since the previous review, five studies that specifically explore AP and pain outcomes have been published [20–24].

### Non-local effects

Flynn et al. [24] demonstrated that there was no relationship between the presence of an AP when receiving SMT and outcomes in patients with LBP in the short term. In addition, Sillevis and Cleland [21] and Cleland et al. [22] reported similar results for patients with neck pain in the short-term. As support of these conclusions, Nim et al. [31] published a systematic review that questioned the concept of segmental specificity on pain outcomes suggesting non-local effects at play, with none of the included studies detecting significant differences in outcome measurements including pain between clinician-determined “correct” vertebral level and surrounding vertebral levels. Such non-local effects support the absence of association between pain reduction and localized ‘pops’, as historically envisaged by early chiropractic theories where the audible sound was originally seen as a result of specific vertebra returning to ‘position’. [32]

Furthermore, in support of non-local effects, Cleland et al. [22] state it may not be realistic to isolate the pop to the target segment. Therefore, they did not attempt to identify if the recorded pop was coming from the segment targeted with the thrust manipulation. It raises questioning of the specificity in terms of location of cavitation regarding the target level of the applied manipulation. Another study by Nim et al. [33] regarding the importance of selecting the “correct” site to apply SMT when treating spinal pain found no difference in reported low back pain intensity when comparing SMT applied to the most painful vertebra or the most stiff vertebra. In support of this, regarding the AP, Beffa and Mathews [34] concluded that the location of cavitation sounds does not appear to be associated with the targeted joints. These findings contradict previous studies in terms of the importance of obtaining an AP [35], as these pops are frequently produced by non-targeted joints as well. Additionally, multiple noises were observed to occur during SMT in the five studies. The common occurrence of multilevel cavitation is in accordance with reports of other studies [36, 37]. Nim et al. [31] suggest positive changes observed after SMT may be unrelated to targeting vertebral sites and might be better explained by mechanisms ranging from neuromuscular or biomechanical interactions, such as functional changes in biomechanical chain and spinal region interdependence [38], to contextual factors modulating pain through cognitively higher interpretations of treatment effects by patients.

Interestingly, Bialosky et al. [20], using a classic pain experimental set up for patients without spinal pain, found that hypoalgesia as measured using heat as external pain stimuli was associated with spinal manipulation in that there was a reduction in perceived pain post manipulation, but that an AP is not required for this effect to be generated. Even though the observed trend by Bialosky et al. [20] of greater hypoalgesia to temporal summation in the lower extremity with the presence of an AP was not statistically significant, the moderate effect size may still indicate potential importance due to underpowering of the study. The only evidence found in support of direct physiological effects of the AP during SMT is described by Clark et al [39]. who reported reduction in erector spinae muscle spindle stretch reflex activity occurred only when SMT was accompanied by an AP. However, this study included only twenty participants, both symptomatic and asymptomatic, and focused on the mechanics of SMT utilizing neurophysiologic assessment techniques which did not include measuring pain outcomes.

### Contextual factors

Beyond local neuromechanical treatment effects, clinical improvement during chiropractic care might be more fully understood using known mechanisms by which contextual factors within therapeutic encounters impact top-down pain modulation [14]. Increasingly, research that acknowledges the importance of such elements including the characteristics of the treatment on clinical outcomes is emerging [40]. Indeed, recent reviews suggests that these factors are very likely important modulators of outcomes in manual therapeutic approaches to pain [41, 42]. In this regard, none of the studies included in this review discussed the potential meaning to the patient of hearing APs during SMT.

In part of the Contextually Aided Recovery (CARe) model [14], the authors speculate that the degree of physical invasiveness of the therapeutic intervention may increase the impact of contextual factor driven analgesia. These authors hypothesize that increasing patient perceived invasiveness from simple touch through manipulation, injection and on to surgery may have increasingly powerful impact on top-down pain modulation mechanisms through increased expectation of therapeutic benefit. In this regard, it might be posited that SMT with an AP can be perceived as more invasive to patients than SMT without an AP. Interestingly, this is not borne out by this review as SMT with AP does not seem to have a stronger hypoalgesic effect compared to SMT without AP and this idea may deserve further investigation.

It is possible that the AP sound has a psychological effect, not only affecting the patient but also affecting the chiropractor and when patient’s expectations of hearing an AP during SMT are not fulfilled it may have a negative effect on the clinical outcome [28]. In a qualitative study on chiropractic patient’s personal perception of the AP [43] participants generally supported the argument that they experienced a release associated with the AP but the majority of patients considered the AP rather unnecessary for successful treatment. However, a cross-sectional online survey on patients receiving thrust manipulation given by a larger spectrum of manual therapists (such as physiotherapists, osteopaths, manual medicine physicians and chiropractors) [44] reported a belief that the presence of the AP was related to the effectiveness of thrust manipulation. Additionally, a high percentage of the patients had beliefs about thrust manipulation and the underlying mechanisms producing an AP that were inconsistent with current literature. Differences in results could possibly be explained by the different nationalities of the patients (United Kingdom and Italy) and the presence of different backgrounds of the clinicians (specifically chiropractors and manual therapists). It appeared that the practitioner’s opinion of the SMT also had an impact on the participant’s perceptions [43]. When the practitioner showed personal satisfaction with the delivered SMT, the participant reported feeling that this was a guarantee of successful treatment. Modern understanding of the types of contextual cues present in therapeutic encounters suggests potential explanations of such phenomena around reduction in the confidence of the practitioner (Practioner characteristics) or impacting the legitimacy of a therapeutic explanation that emphasises manipulation as the singular cause of clinical outcomes (Treatment characteristics) [45].

Interestingly, Van Geyt et al. [37] observed the frequency of cavitation production to increase the more years the clinician had been in practice. This is linked with Williams and Cuesta-Vargas [46], who observed that the presence of cavitation is associated with greater thrust accelerations, which are more commonly achieved by more practiced clinicians. Hence for a more experienced practitioner it might be easier to achieve an AP and therefore be more confident in showing personal satisfaction with the delivered treatment.

### Limitations

Limitations of this review include some common methodological problems in included studies, such as lack of blinded assessment which could weaken the evidence in these experimental studies. To separate physiologic effects from effects based on subject expectations, subjects must be fully and convincingly blinded to their treatment. Unfortunately, true blinding has proven to be very difficult to achieve regarding SMT; placebo physical interventions often differ too much to the physical experience of SMT making it potentially straightforward for the patient to identify shams.

Secondly, although this review found that an AP does not have an effect on perceived pain regardless of the area of the spine manipulated, we do not know if this is generalizable to extra spinal locations such as the wrist or ankle joint.

In addition, the search for this study resulted in only 69 results, which may be considered a somewhat low number for a systematic review. The possibility remains that our focused search terms did not identify studies that reported relevant results as peripheral to their main objective, and therefore may not have been captured. Alternatively, such studies may just be uncommon due to methodological challenges and therefore genuinely low in frequency. Lastly, despite the included studies being classified medium to high quality, one study [20] only included 40 participants, which is a small sample size for quantitative research [47]. Therefore, this former study’s conclusions should be interpreted with caution as generalizable to a wider population. However, whilst overall, considerable heterogeneity existed between participants regarding pain site and intensity, all included studies pointed in a similar direction, implying potential increased confidence in the conclusions presented here.

## Conclusions

In summary, there is currently an absence of evidence that supports a relationship between the presence of an audible pop during the delivery of SMT and pain outcomes. So, whilst it is still unclear as to the factors that underly clinical improvement associated with approaches that include SMT, this aspect of SMT practice does not seem to be an important factor for the hypoalgesic effect. In terms of clinical practice then, this review supports the notion that clinicians need not overemphasize the presence of a perceived AP as an indicator of successful treatment. However, noting that some practitioners and patients still consider this aspect an important part of the SMT experience, further research would be helpful in fully comprehending the contribution to the perceived meaning of this phenomenon to both patients and practitioners.

## Supplementary Information

**Additional file 1:** PRISMA 2020 Checklist.

**Additional file 2:** Search Strategy Conducted in CINAHL.

## Data Availability

Data sharing is not applicable to this article as no datasets were generated or analyzed during the current study.

## Abbreviations

AP: Audible pop
HVLA: High-velocity low-amplitude
HVT: High velocity thrust
LBP: Low back pain
NPRS: Numeric pain rating scale
PRISMA: Preferred reporting items for systematic reviews and meta-analysis
ROM: Range of motion
SMT: Spinal manipulative treatment
VAS: Visual analog scale
