# Supplementary material for: Impact of audible pops associated with spinal manipulation on perceived pain: a systematic review
Source: Chiropr Man Therap. 2022 Oct 4;30:42. doi: 10.1186/s12998-022-00454-0 (PMC9531394; doi:10.1186/s12998-022-00454-0)
Supplement: Supplementary file 2 — Additional file 2: Search Strategy Conducted in CINAHL. [file 12998_2022_454_MOESM2_ESM.docx]

**Appendix 1: Search Strategy Conducted in CINAHL**

| S19 | S6 AND S14 AND S17 | **Expanders** - Apply equivalent subjects  **Search modes** - Find all my search terms | **View Results** (14)  **View Details**  [**Edit**](http://web.b.ebscohost.com/Legacy/Views/UserControls/EHOST/) |  |
| --- | --- | --- | --- | --- |
|  | S17 | S15 OR S16 | **Expanders** - Apply equivalent subjects  **Search modes** - Find all my search terms | **View Results** (321,524)  **View Details**  [**Edit**](http://web.b.ebscohost.com/Legacy/Views/UserControls/EHOST/) |
|  | S16 | "Pain" | **Expanders** - Apply equivalent subjects  **Search modes** - Find all my search terms | **View Results** (321,524)  **View Details**  [**Edit**](http://web.b.ebscohost.com/Legacy/Views/UserControls/EHOST/) |
|  | S15 | (MH "Pain") | **Expanders** - Apply equivalent subjects  **Search modes** - Find all my search terms | **View Results** (75,046)  **View Details**  [**Edit**](http://web.b.ebscohost.com/Legacy/Views/UserControls/EHOST/) |
|  | S14 | S7 OR S8 OR S9 OR S10 OR S11 OR S12 OR S13 | **Expanders** - Apply equivalent subjects  **Search modes** - Find all my search terms | **View Results** (5,379)  **View Details**  [**Edit**](http://web.b.ebscohost.com/Legacy/Views/UserControls/EHOST/) |
|  | S13 | "Spinal Manipulation" | **Expanders** - Apply equivalent subjects  **Search modes** - Find all my search terms | **View Results** (1,311)  **View Details**  [**Edit**](http://web.b.ebscohost.com/Legacy/Views/UserControls/EHOST/) |
|  | S12 | "High Velocity Low Amplitude Thrust" | **Expanders** - Apply equivalent subjects  **Search modes** - Find all my search terms | **View Results** (43)  **View Details**  [**Edit**](http://web.b.ebscohost.com/Legacy/Views/UserControls/EHOST/) |
|  | S11 | "High Velocity Low Amplitude Adjustment" | **Expanders** - Apply equivalent subjects  **Search modes** - SmartText Searching | **View Results** (5,598)  **View Details**  [**Edit**](http://web.b.ebscohost.com/Legacy/Views/UserControls/EHOST/) |
|  |  |  |  |  |
|  | S10 | "Chiropractic Adjustment" | **Expanders** - Apply equivalent subjects  **Search modes** - Find all my search terms | **View Results** (2,820)  **View Details**  [**Edit**](http://web.b.ebscohost.com/Legacy/Views/UserControls/EHOST/) |
|  | S9 | "Chiropractic Manipulation" | **Expanders** - Apply equivalent subjects  **Search modes** - Find all my search terms | **View Results** (2,920)  **View Details**  [**Edit**](http://web.b.ebscohost.com/Legacy/Views/UserControls/EHOST/) |
|  | S8 | "Spinal Manipulative Therapy" | **Expanders** - Apply equivalent subjects  **Search modes** - Find all my search terms | **View Results** (490)  **View Details**  [**Edit**](http://web.b.ebscohost.com/Legacy/Views/UserControls/EHOST/) |
|  | S7 | (MH "Manipulation, Chiropractic") | **Expanders** - Apply equivalent subjects  **Search modes** - Find all my search terms | **View Results** (4,262)  **View Details**  [**Edit**](http://web.b.ebscohost.com/Legacy/Views/UserControls/EHOST/) |
|  | S6 | S1 OR S2 OR S3 OR S4 OR S5 | **Expanders** - Apply equivalent subjects  **Search modes** - Find all my search terms | **View Results** (77)  **View Details**  [**Edit**](http://web.b.ebscohost.com/Legacy/Views/UserControls/EHOST/) |
|  | S5 | "Joint Cracking" | **Expanders** - Apply equivalent subjects  **Search modes** - Find all my search terms | **View Results** (23)  **View Details**  [**Edit**](http://web.b.ebscohost.com/Legacy/Views/UserControls/EHOST/) |
|  | S4 | "Joint Cavitation" | **Expanders** - Apply equivalent subjects  **Search modes** - Find all my search terms | **View Results** (12)  **View Details**  [**Edit**](http://web.b.ebscohost.com/Legacy/Views/UserControls/EHOST/) |
|  | S3 | "Audible Pop" | **Expanders** - Apply equivalent subjects  **Search modes** - Find all my search terms | **View Results** (17)  **View Details**  [**Edit**](http://web.b.ebscohost.com/Legacy/Views/UserControls/EHOST/) |
|  | S2 | "Audible Release" | **Expanders** - Apply equivalent subjects  **Search modes** - Find all my search terms | **View Results** (53)  **View Details**  [**Edit**](http://web.b.ebscohost.com/Legacy/Views/UserControls/EHOST/) |
|  | S1 | (MH "Audible Release (Joint)") |  |  |
